# Secondary osteoporosis prevention: three-year outcomes from a Fracture Liaison Service in elderly hip fracture patients

**DOI:** 10.1007/s40520-024-02761-3

**Published:** 2024-05-05

**Authors:** David González-Quevedo, Carolina Rubia-Ortega, Adriana Sánchez-Delgado, Diego Moriel-Garceso, Juan-Manuel Sánchez-Siles, Manuel Bravo-Bardají, David García-de-Quevedo, Iskandar Tamimi

**Affiliations:** 1 Department of Orthopedic Surgery and Traumatology, Regional University Hospital of Málaga, Carlos Haya Avenue, 29010 Málaga, Spain; 2 Department of Surgical Specialties, Biochemistry and Immunology, School of Medicine, University of Málaga, Málaga, Spain

**Keywords:** Fracture liaison service, Hip fractures, Osteoporosis, Mortality, Re-fracture.

## Abstract

**Background:**

Hip fractures are the most serious fragility fractures due to their associated disability, higher hospitalization costs and high mortality rates. Fracture Liaison Service (FLS) programs have enhanced the management of osteoporosis-related fractures and have shown their clinical effectiveness.

**Aims:**

To analyze the effect of the implementation of a FLS model of care over the survival and mortality rates following a hip fracture.

**Methods:**

We conducted a prospective cohort study on patients over 60 years of age who suffered a hip fracture before and after the implementation of the FLS in our center (between January 2016 and December 2019). Patients were followed for three years after the index date. Mortality, complications and refracture rates were compared between the two groups using a Multivariate Cox proportional hazard model.

**Results:**

A total of 1366 patients were included in this study (353 before FLS implementation and 1013 after FLS implementation). Anti-osteoporotic drugs were more frequently prescribed after FLS implementation (79.3% vs 12.5%; p < 0.01) and there was an increase in adherence to treatment (51.7% vs 30.2%; p < 0.01). A total of 413 (40.8%) patients after FLS implementation and 141 (39.9%) individuals before (p = 0.47) died during the three-years follow-up period. A second fracture occurred in 101 (10.0%) patients after FLS implementation and 37 (10.5%) individuals before (p = 0.78). Patients after the implementation of the FLS protocol had a lower all cause one-year mortality [adjusted Hazard Ratio (HR) 0.74 (0.57–0.94)] and a decreased risk of suffering a second osteoporotic fracture [adjusted HR 0.54 (0.39–0.75) in males and adjusted HR 0.46 (0.30–0.71) in females].

**Conclusions:**

The implementation of a FLS protocol was associated with a lower all-cause one-year mortality rate and a higher survivorship in elderly hip fracture patients. However, no three-year mortality rate differences were observed between the two groups. We also found a reduction in the complication and second-fracture rates.

## Introduction

Osteoporosis is a metabolic disease characterized by a low bone mass and altered microarchitecture resulting in a higher risk of suffering fragility fractures [1]. In 2019, 25.5 million women and 6.5 million men were predicted to have osteoporosis in the European Union, the United Kingdom and Switzerland, and 4.3 million of new fragility fractures were calculated [2].

Hip fractures are the most serious fragility fracture due to their significantly high morbidity and mortality rates [3]. Moreover, the economic cost associated with the treatment of hip fractures is very high and it has continually increased in recent years [4]. In Italy, the overall costs associated with the treatment of hip fractures raised from 735 to 961 million Euros from 2000 to 2014 (+ 30.74%) [5].

The fracture liaison service (FLS) care is considered the best organizational approach for secondary fracture prevention [6]. This model of care has been reported to be associated with a significantly lower probability of subsequent fractures and mortality rates [7].

Our research team conducted two previous studies in which we observed that the implementation of the FLS improved the one-year overall survival of patients with hip fractures without resulting in a reduction in secondary fracture rates [8, 9]. Accordingly, this study is a continuation of our previous research, in which we have prolonged the post-FLS-implementation follow-up period to three years, in order to look for any changes in the fracture rates. We hypothesize that the use of an intensive FLS model of care in our institution could improve the survivorship of elderly hip fracture patients without a reduction of second fragility fracture. Therefore, the primary aim of this study was to analyze the effect of the FLS model over the survival and mortality rates following a hip fracture. The secondary aim was to determine the risk of suffering a second osteoporotic fracture and the adherence to treatment.

## Material and methods

### Study design

A prospective cohort study was conducted on hip fracture patients over 60 years, who were treated in our institution between January 2016 and December 2019. The first group of patients were diagnosed between January 2016 and December 2016, before the implementation of the FLS. Patients in the second group were diagnosed between January 2017 and December 2019, after the implementation of the FLS protocol. Overlapped patients were excluded from FLS group. Patient’s records were withdrawn from the regional public Andalusian healthcare system database, which is linked to the national Spanish mortality registry. Patients with pathological fractures (i.e., osteomalacia, Paget’s disease, history of malignancy) were excluded from the study. The following information was collected form our local computerized database: age, gender, American Society of Anesthesiologists (ASA) score, fracture side, fracture type (i.e., femoral neck, trochanteric or subtrochanteric), surgical treatment (i.e., cannulated screws, proximal femoral nail, hemiarthroplasty or total hip replacement), prescriptions and adherence to anti-osteoporotic drugs (i.e., adherent patients were defined as individuals who were prescribed these drugs throughout all the follow-up period). Patients were followed from the date of the initial hip fracture (index date) until death of any cause, or end of the three years follow-up period. Complications (i.e. infection, cut-out, cut-in, aseptic loosening, non-union, dislocation and medical complication), second osteoporotic fractures (i.e., contralateral hip, distal radius, proximal humerus and radiological vertebral fractures), readmissions and reinterventions were considered as secondary outcomes. Subsequent fractures were identified from individual medical records of the regional public healthcare system database. Exposure to drugs used for the treatment of osteoporosis were assessed only if these were used at any point after the index date.

### FLS protocol

Before the implementation of the FLS protocol, patients received a standard fracture care during hospitalization. Patients had an out-patient visits 1 month after the index date. Further visits were scheduled depending on the patients’ evolution and surgeons’ preferences.

On the other hand, FLS patients followed the protocol previously described by our institution [8]. Briefly, patents underwent a series of laboratory tests during the in-patient period (including a basic biochemistry test, calcium serum levels, albumin, vitamin D, among others). In addition, mobility was assessed using functional ambulation categories (FAC) scale and autonomy was evaluated using Barthel’s scale. During the in-patient period, medical comorbidities were treated and physical therapy was started. All patients as well as their respective carers received an exercise program. Osteoporotic treatment was started on discharge according to the European guidance for diagnosis and management of osteoporosis, and the recommendations of the International Osteoporosis Foundation (IOF) [10, 11] and the Spanish Society of Rheumatology [12], which include calcium and vitamin D supplements. Oral bisphosphonates (i.e. alendronic acid and risedronate) were not prescribed in patients with renal impairment or gastric intolerance. In those subject subcutaneous denosumab was prescribed. Teriparatide was prescribed for cases of severe osteoporosis, defined as patients having two or more major fragility fractures or those with one major fragility fracture and a T-score of -3.0 or lower on the Bone Mineral Density test. Patient had outpatient visits appointments after 1, 6 and 12 months from the index date. In these visits, Barthel’s and FAC scales were repeated, fracture care was received, and potential health issues were identified.

### Statistical analysis

Data were analyzed with SPSS 24.0 software (SPSS Inc., Chicago, IL, USA), and G*power 3.1.9.6 (Universität Kiel, Germany). Categorical variables were presented as absolute values and percentages. Means were presented with their corresponding standard deviations (SD). The distribution of the continuous variables was assessed using the Shapiro–Wilk test. Differences between the demographic features were analyzed using t-student and Chi square tests. Patient survival was determined using a Kaplan–Meier survivorship analysis. Two different analyses were performed. First, a survival analysis where the outcomes were either death or end of the 36-months follow-up period, here patients lost to follow-up were censored. In the second analysis, mortality, any complication and second osteoporotic fracture rates were compared between the two groups using a Multivariate Cox proportional hazard model adjusted to potential confounders: age, type of fracture and ASA score. Post-hoc power analyses for students-t and Chi square tests, were used with an α- error probability of 0.05. Mortality intervals were cumulative (e.g., the mortality at 2 years included the mortality from the index date to the end of the second year). The proportionality hazard assumption was test using and Omnibus test, computing time-dependent covariates and log-minus-log graphs. Variables with a p-value < 0.05 were considered time dependent. The mortality and time dependence were considered when on calculating for second fractures and complication rates.

## Results

A total of 1366 patients fulfilled the inclusion–exclusion criteria (432 males and 934 females) (Table 1). The mean patient age was 82.34 ± 7.84 years (i.e., 82.27 ± 8.21 before FLS implementation, and 82.36 ± 7.71 after FLS implementation, p = 0.32). Anti-osteoporotic treatment was given in 44 cases (12.5%) before FLS implementation; compared to 803 (79.3%) patients after FLS implementation, odds ratio (OR) of 11.46 (8.52–15.41) (p < 0.01). There were no statistically significant comorbidity differences between the two groups (ASA scale 2.58 ± 0.70 vs. 2.61 ± 0.67, p = 0.25) (Table 1). However, the overall survival was higher after the implementation of the FLS protocol compared to the period before its implementation (i.e., 802.63 ± 413.80 vs. 823.93 ± 389.20 days respectively, p = 0.01, power 95%) (Table 1). In total, 554 patients (40.6%) died during the three-years follow-up period: 141 patients (39.9%) before FLS implementation and 413 patients (40.8%) after FLS implementation group (p = 0.79) (Table 1).

**Table 1:** Patient demographic and clinical features

Parameter	Before FLS implementation (n = 353)	After FLS implementation (n = 1013)	P value
Age, years	82.27 ± 8.21	82.36 ± 7.71	0.32
Gender			
Male	71 (20.1)	361 (35.6)	< 0.01*
Female	282 (79.9)	652 (64.4)	
Side			
Left	172 (48.7)	519 (51.2)	0.41
Right	181 (51.3)	494 (48.8)	
Fracture type			
Femoral neck	149 (42.2)	391 (38.6)	0.46
Trochanteric	175 (49.6)	540 (53.3)	
Subtrochanteric	29 (8.2)	82 (8.1)	
ASA	2.58 ± 0.70	2.61 ± 0.67	0.25
0	0 (0.0)	2 (0.2)	0.26
1	6 (1.7)	7 (0.7)	
2	175 (49.6)	475 (46.9)	
3	135 (38.2)	432 (42.6)	
4	37 (10.5)	97 (9.6)	
Anti-Osteoporotic treatment rate	44 (12.5)	803 (79.3)	< 0.01*
Initiated at hospitalization	22 (6.2)	556 (55.0)	< 0.01*
Initiated at out-patient clinic	22 (6.2)	244 (24.1)	< 0.01*
Anti-Osteoporotic Drugs			
Oral bisphosphonates	22 (50.0)	673 (83.8)	< 0.01*
Denosumab	14 (31.8)	61 (7.6)	
Teriparatide	8 (18.2)	69 (8.6)	

Data are presented as No. (%) or mean ± SD

*FLS* Fracture Liaison Service; *ASA* American Society of Anesthesiologists physical status classification system.

*Statistically significant

A total of 138 patients (10.1%) suffered a second osteoporotic fracture: 37 patients (10.5%) before FLS implementation and 101 patients (10.0%) after FLS implementation (p = 0.78), from which 57 (4.2%) were contralateral hip fractures [i.e., 15 (3.9%) and 42 (3.8%) in each group, respectively] (Table 2). There was a higher adherence to treatment after FLS implementation (51.7%) compared to adherence before its application (30.2%) (p < 0.01) (Table 2). In this sense, the treatment discontinuation rates were 47.0% for bisphosphonates, 56.8% for denosumab and 62.3% for teriparatide.

**Table 2:** Outcomes and complications

Parameter	Before FLS implementation (n = 355)	After FLS implementation (n = 1044)	P value
Three-year mortality rate	141 (39.9)	413 (40.8)	0.79
One-month mortality rate	11 (3.1)	22 (2.2)	0.32
First-year mortality rate	77 (22.5)	187 (18.9)	0.14
Second-year mortality rate	24 (9.1)	107 (13.3)	0.07
Three-year mortality rate	29 (12.1)	97 (13.9)	0.48
Survival, days	802.63 ± 413.80	823.93 ± 389.20	0.01*
Second fracture rate	37 (10.5)	101 (10.0)	0.78
Hip fracture	15 (3.9)	42 (3.8)	0.46
Other fractures	22 (6.2)	59 (5.6)	
Adherence to treatment	13 (30.2)	416 (51.7)	< 0.01*
Complications	27 (7.6)	61 (6.0)	0.28
Cut-out	1 (0.3)	10 (0.9)	0.1
Medical complication	14 (3.9)	21 (2.0)	
Readmission	10 (2.8)	25 (2.5)	0.71
Reintervention	14 (3.9)	39 (3.7)	0.92

Data are presented as No. (%) or mean ± SD

*FLS* Fracture Liaison Service; *ASA* American Society of Anesthesiologists physical status classification system.

* Statistically significant

On the other hand, there were no statistical differences regarding complications [i.e., 27 (7.6%) vs 61 (6.0%), p = 0.28], readmission [i.e., 10 (2.8%) vs 25 (2.5%), p = 0.71] and reintervention [i.e., 15 (3.9%) vs 39 (3.7%), p = 0.92] rates between groups (Table 2).

**Fig. 1:**
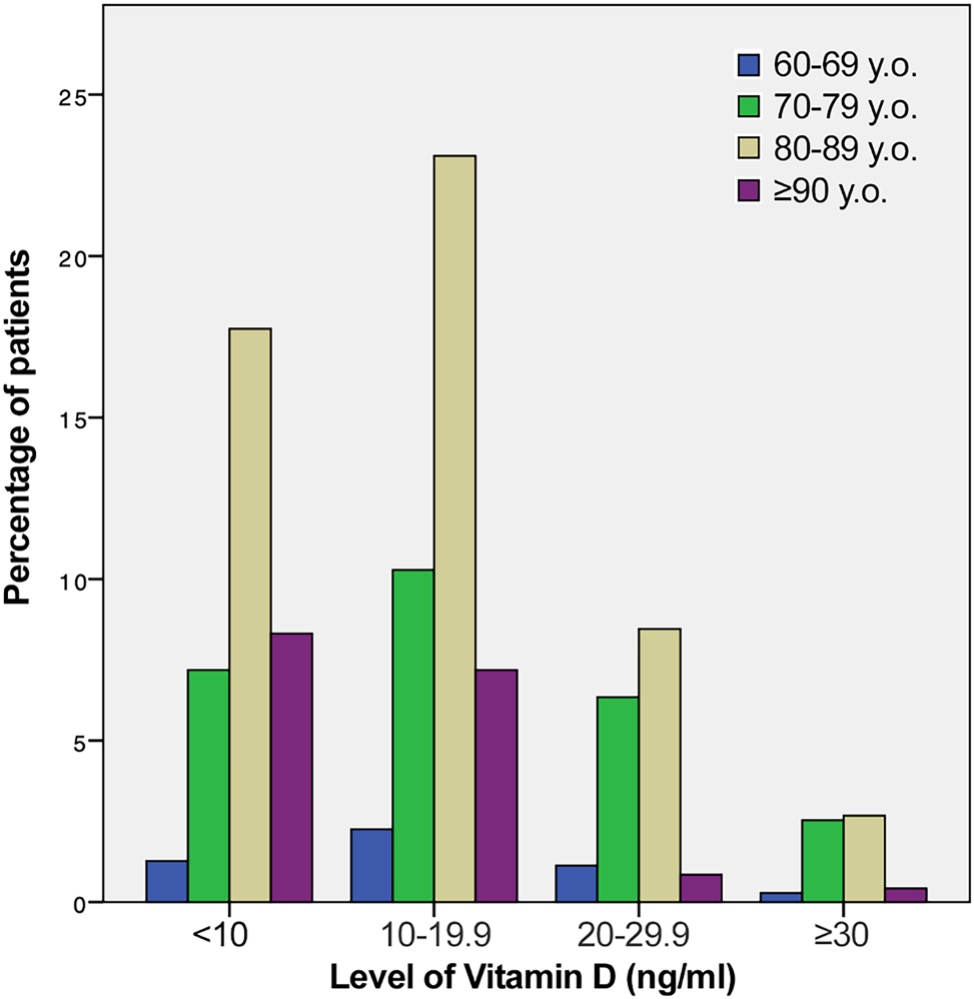
Percentage of patients according to level of vitamin D (ng/ml) (< 10 ng-dL severe deficiency, 10–19.9 ng-dL moderate deficiency, and 20–29 ng-DL relative deficiency), classified by age (years old)

There was a widespread deficiency of albumin (2.63 ± 0.61 g/dl) and vitamin D (15.09 ± 10.57 ng/dl) levels in our patients. The following cut-off points for vitamin D deficiency were determined: < 10 ng-dL severe deficiency, 10–19.9 ng-dL moderate deficiency, and 20–29 ng-DL relative deficiency. These levels worsened as the age of the patients increased (Table 3). Moreover, we found a deficiency of Vitamin D in 94.1% of these hip fracture patients (i.e., 34.5% with a severe deficiency, 42.8% with a moderate deficiency and 16.8% with a relative deficiency) (Fig. 1).

**Table 3:** Routine blood test results

	**Albumin (g/dl)**	**Calcium (mg/dl)**	**Vitamin D (ng/dl)**
	2.63 ± 0.61	8.14 ± 0.76	15.02 ± 10.59
Gender			
Male	2.66 ± 0.64	8.17 ± 0.74	15.83 ± 9.49
Female	2.62 ± 0.59	8.12 ± 0.78	14.52 ± 11.20
Age, years			
60–69	3.12 ± 1.08	8.41 ± 1.18	15.65 ± 8.05
70–79	2.73 ± 0.60	8.24 ± 0.75	16.86 ± 9.37
80–89	2.58 ± 0.59	8.08 ± 0.77	14.75 ± 9.60
90–99	2.55 ± 0.43	8.10 ± 0.58	12.61 ± 14.89

Data are presented as mean ± SD

**Fig. 2:**
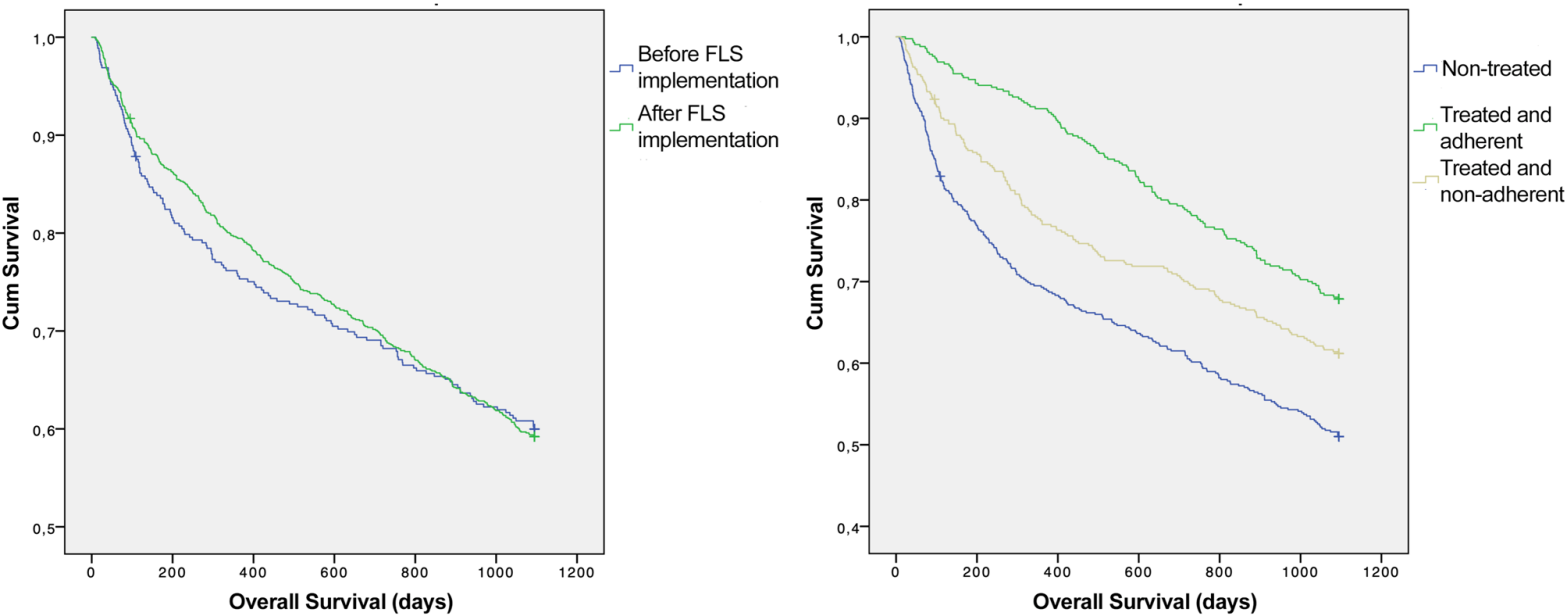
Kaplan–Meier estimates of survival time for patients with hip fractures: before and after FLS implementation (left) and non-treated patients vs. treated and adherent patients vs. treated and non-adherent patients (right)

The Cox proportional hazards model showed a significant adjusted one-year hazard ratio (HR) for all-cause mortality of 0.74 (0.58–0.96) [0.57 (0.37–0.88) in male patients and 0.77 (0.56–1.05) in female patients] after the implementation of the FLS protocol compared with individuals treated before the implementation of the FLS protocol (Table 4, Fig. 2). The Cox proportional hazards model also showed a significant reduction in the complication rate between the FLS group compared with patients before FLS implementation [adjusted HR 0.62 (0.46–0.84)]; 0.37 (0.23–0.60) in male patients, and 0.75 (0.51–1.10) in female patients] (Table 4). In addition, statistical differences in the risk of suffering a second osteoporotic fracture were found between patients treated before FLS implementation and individuals included in the FLS protocol [adjusted HR 0.54 (0.39–0.75)]; 0.80 (0.47–1.35) in male patients and 0.46 (0.30–0.71) in female patients] (Table 4).

**Table 4:** Multivariable cox regression analysis on mortality and second fracture rates: before FLS-implementation vs. after FLS implementation

Females	Before FLS implementation (n = 282)	After FLS implementation (n = 652)	Crude HR	Adjusted HR
One-month mortality rate	7 (2.5)	14 (2.1)	0.86 (0.35–2.13)	0.75 (0.30–1.87)
One-year mortality rate	62 (22.0)	118 (18.1)	0.81 (0.59–1.10)	0.77 (0.56–1.05)
Two-year mortality rate	82 (29.1)	185 (28.4)	0.96 (0.74–1.24)	0.93 (0.72–1.21)
Three-year mortality rate	105 (37.2)	247 (37.9)	1.00 (0.78–1-26)	0.98 (0.78–1.23)
Any complication	20 (7.1)	43 (6.6)	0.74 (0.51–1.09)	0.75 (0.51–1.10)
Second fracture	30 (10.6)	72 (11.0)	0.49 (0.32–0.74)*	0.46 (0.30–0.71)*

Total	Before FLS implementation (n = 353)	After FLS implementation (n = 1013)	Crude HR	Adjusted HR
One-month mortality rate	4 (5.6)	8 (2.2)	0.39 (0.12–1.28)	0.36 (0.11–1.22)
One-year mortality rate	28 (39.4)	90 (24.9)	0.56 (0.39–0.86)*	0.57 (0.37–0.88)*
Two-year mortality rate	32 (45.1)	131 (36.3)	0.70 (0.48–1.04)	0.70 (0.47–1.03)
Three-year mortality rate	35 (49.3)	166 (46.0)	0.81 (0.56–1.16)	0.80 (0.56–1.15)
Any complication	7 (9.9)	18 (5.0)	0.40 (0.25–0.64)*	0.37 (0.23–0.60)*
Second fracture	7 (9.9)	29 (8.0)	0.77 (0.46–1.30)	0.80 (0.47–1.35)

Males	Before FLS implementation (n = 71)	After FLS implementation (n = 361)	Crude HR	Adjusted HR
One-month mortality rate	11 (3.1)	22 (2.2)	0.69 (0.34–1.42)	0.64 (0.31–1.32)
One-year mortality rate	90 (25.5)	208 (20.5)	0.78 (0.61–1.00)	0.74 (0.58–0.96)*
Two-year mortality rate	114 (32.3)	316 (31.2)	0.93 (0.76–1.16)	0.92 (0.74–1.14)
Three-year mortality rate	140 (39.7)	413 (40.8)	1.00 (0.83–1.21)	0.98 (0.81–1.19)
Any complication	27 (7.6)	61 (6.0)	0.62 (0.46–0.84)*	0.62 (0.46–0.84)*
Second fracture	37 (10.5)	101 (10.0)	0.56 (0.41–0.78)*	0.54 (0.39–0.75)*

Values adjusted to age, type of fracture, American Society of Anesthesiologists (ASA) score

Data are presented as No. (%)

*FLS* Fracture Liaison Service; *HR* hazard ratio

*Statistically significant

## Discussion

In the present study, we found that the implementation of a FLS model of care showed a significantly lower adjusted one-year hazard ratio for all-cause mortality; this finding was consistent with our previous research [8, 9]. Moreover, a recent study showed that the introduction of a FLS in a single institution in Netherlands was associated with a lower mortality risk in the first 3 years [13]. Another cohort study revealed that patients who received anti-osteoporotic drugs for more than 1, 2, and 3 years also exhibited proportional reductions in all-cause mortality and a longer duration of the treatment was related with a lower mortality [14]. In general, the systematic reviews that evaluate the clinical impact of FLS implementation suggested the reduction of mortality among these patients [7, 15].

The reduction in the mortality following a hip fragility fracture has been attributed to the use of bisphosphonate, as well as non-bisphosphonate medications used for the treatment of osteoporosis [16]. Previous research has found that women with osteopenia who received zoledronic acid had fewer cardiovascular events, cancers and mortality rates [17]. In addition, zoledronic acid injections after surgery in extracapsular hip fracture patients relieve the pain, reduce the refracture incidence and improve bone metabolism and bone mineral density [18]. On the other hand, it seems that patients with calcium-vitamin D supplementation in combination with osteoporosis drugs had a lower risk of both subsequent fractures and all-cause mortality based on the data from five Italian Local Health Units [19]. Moreover, a higher five-years survival has been found among patients who underwent hemiarthroplasty for hip fracture and received osteoporosis treatment [20].

On the other hand, we found that there were no differences between groups in the mortality rate after one year of follow up. Moreover, our three-years mortality rate (i.e., 40%) is very similar to another report performed in Turkey in which, authors recommend a multidisciplinary approach to hip fracture patients due its associated comorbidities [21]. The significant reduction of the adjusted one-year mortality rate may be explained by the improvement in the care for these patients in the public health system which may has led to a significant decline in one-year mortality in the last decade [22]. Interestingly, a study performed in Italy between 2000 and 2015 showed an increase in the age, institutionalization and comorbidities of hip fracture patients. However, the length of hospital stay was lower, and no differences in the 30-days and one-year mortality rates were observed between the different groups [23]. In this sense, another Finnish study with a 14-years of follow-up, suggested that the ASA score, body max index and age were the most predictive factors for one-year and fourteen-year survival [24]. Interestingly, our Kaplan–Meier analysis showed a higher survival of those treated but non-adherent patients. This can be explained by a meta-analysis in which the included studies reported a higher adherence at older ages than younger ages [25].

Moreover, our results suggested that both hypoproteinemia and Vitamin D deficiency were very common among elderly hip fracture patients. Preoperative hypoalbuminemia and an increased age has been reported as independent risk factors for two-years mortality rate according to a recent report from Shanghai [26]. In addition, the presence of comorbidities has been also associated with higher mortality rates in hip fracture patients. Therefore, the use of the Charlson Comorbidity Index or ASA score may be useful tools to predict the two-years mortality rates in elderly hip fracture patients [27]. However, novel research, suggests that Vitamin D deficiency is not associated with all-cause 6-month mortality after hip fracture surgery, being more determinants factors the presence of other comorbidities and the patient’s functional status [28]. On the other hand, a recent meta-analysis has shown that 25-hydroxivitamin D has been related with an increase in the incidence of delirium in hospitalized patients [29].

Another interesting finding of our study is that we found statistical differences between groups regarding the adjusted second fragility fracture rate after three years of follow-up. Moreover, a previous study showed a reduction of up to 30% in the risk of any refracture rates in patients presenting to an Australian hospital with a FLS, when compared to a similar hospital without a FLS protocol [30]. These results were similar to another report that showed a reduction in the incidence of all refractures in patients with vertebral compression fractures that followed a FLS protocol [31]. In addition, the FRAME study which enrolled more than 7000 patients demonstrated that romosozumab therapy was also associated with rapid and large reductions in clinical vertebral fracture risk compared with placebo [32]. It seems that the real impact of FLS programs on subsequent fracture rates is uncertain due to the heterogeneous designs and populations of the different studies [33]. In fact, our previous research performed in a single institution never showed a reduction of secondary fracture rates [8, 9]. Our previous results also differ from a recent meta-analysis performed on > 80.000 patients that showed the benefit of osteoporosis treatment in postmenopausal women by reducing the refracture risk. This effect was mostly independent of baseline risk indicators [34]. This can be explained by microstructural studies which suggest that the predictive value of the bone mineral density decreases in the older population [35, 36]. Nevertheless, there is a global consensus for starting pharmacologic therapy for osteoporosis to people with a hip fracture to reduce the risk of additional fractures [37].

Finally, we found that the implementation of a FLS model of care reduced surgical-related complications (i.e. infection, cut-out, cut-in, aseptic loosening, non-union, dislocation and medical complication). These results highlight the potential cost-effectiveness of this model of care [38].

To the best of our knowledge, this cohort study is the first to report a reduction off all-cause fist-year mortality and complication rates after the implementation of an intensive FLS model with a reduction of the adjusted second fracture rate of patients with hip fractures in a single hospital. The prospective design of this study could decrease the introduction of significant selection bias. Moreover, the results of this study were adjusted to potential confounders, using a Cox proportional hazard model. Nevertheless, our study is also subjected to several limitations. Firstly, this was an observational study, and further randomized controlled studies would be recommended to determine the real efficacy of the FLS protocol. However, these studies could involve ethical issues because second fracture prevention protocols have showed better outcomes compared with the traditional management of hip fracture patients. Secondly, the sample size of the pre-implementation cohort population was relatively smaller than the FLS cohort population; however, the statistical power of our results was near to 95%. Finally, the follow-up period in this study was limited to three years that could be a relatively short period for the prevention of second fractures. Therefore, the continuation of further studies, with longer follow-up periods would be recommended to evaluate the long-term mortality and second fracture rates of the FLS protocols on hip fracture patients.

## Conclusion

The implementation of a FLS protocol in elderly hip fracture patients in our institution was associated with a lower all-cause one-year mortality rate and a higher survivorship. However, no three-year mortality rate differences were observed compared to the traditional care of these patients. On the other hand, we found a reduction of the adjusted complications rate and of the risk of suffering a second fragility fracture after three-years of follow up.

## Data Availability

The data that support the findings of this study are available on request from the corresponding author. The data are not publicly available due to privacy or ethical restrictions.
